# Initial and relapse prodromes in adult patients with episodes of bipolar disorder: A systematic review

**DOI:** 10.1192/j.eurpsy.2019.18

**Published:** 2020-02-12

**Authors:** Nelson Andrade-González, Laura Álvarez-Cadenas, Jerónimo Saiz-Ruiz, Guillermo Lahera

**Affiliations:** 1 Relational Processes and Psychotherapy Research Group, Faculty of Medicine and Health Sciences, University of Alcalá, Alcalá de Henares, Madrid, Spain; 2 Independent Practice, Oviedo, Spain; 3 Ramón y Cajal University Hospital, Madrid, Spain; 4 Faculty of Medicine and Health Sciences, University of Alcalá, Alcalá de Henares, Madrid, Spain; 5 IRyCIS, CIBERSAM, Madrid, Spain

**Keywords:** bipolar disorder, prodrome, systematic review

## Abstract

**Background.:**

Distinguishing prodromes of bipolar disorder (BD) specific to children/adolescents, adults, and elderly patients is essential. The primary objective of this systematic review was to determine initial and relapse prodromes identifying adult patients with BD.

**Methods.:**

PubMed, PsycINFO, and Web of Science databases were searched using a predetermined strategy. A controlled process of study selection and data extraction was performed.

**Results.:**

The 22 articles selected included 1,809 adult patients with BD. Initial prodromes cited most frequently in these studies showed low specificity. Among relapse prodromes cited most frequently, more talkative than usual, increased energy/more goal-directed behavior, thoughts start to race, increased self-esteem, strong interest in sex, increase in activity, and spending too much were identified exclusively before a manic/hypomanic episode, while loss of interest and hypersomnia were detected only before a depressive episode. Initial prodromal phases lasted longer than prodromal relapse phases. In the selected studies, the most used prodrome identification procedure was the clinical interview.

**Conclusions.:**

For adult patients with BD, initial and relapse prodromes of manic, hypomanic, and depressive episodes were identified. It is proposed that the most frequent prodromes found in this review be incorporated into a smartphone app that monitors the functioning of people at risk of BD and patients who have already been diagnosed. Data from this app would constitute a relevant source of big data.

## Introduction

Bipolar disorder (BD) is an affective disorder characterized by the cyclical presence of recurrent manic, hypomanic, and depressive affective episodes. To diagnose type I BD, the patient must have at least one episode of mania; to diagnose type II BD, the patient must have at least one episode of hypomania and at least one major depressive episode [1]. The Global Burden of Disease Study 2013 revealed that, in that year, 48.8 million people in the world suffered from BD, with more prevalence in women and patients aged between 25 and 29 years. Furthermore, among mental disorders, BD ranked fifth in lost years of healthy life [2]. Patients with BD have up to 20–30 times more risk of suicide than the general population [3] and usually have comorbid psychopathology [4]. Merikangas et al. [5] reported that more than 90% of American patients with BD (including those living with subthreshold BD) also had another lifelong Axis I disorder (mainly anxiety disorders according to the Diagnostic and Statistical Manual of Mental Disorders, Fourth Edition [DSM-IV]). On a global scale, Hunt et al. [6] found high comorbidity between BD and substance use disorders in hospital samples and community samples, particularly with alcohol use disorders (mean 30%) and cannabis use disorders (mean 20%). Consequently, early detection of BD and intervention during the prodromal stage can contribute to reducing the burden of the disorder by preventing onset of the complete disorder [2] while reducing comorbidity.

A prodrome is “the period of disturbance which represents a deviation from a person’s previous experience and behavior prior to the development of the florid features of a disorder” [7, p. 556]. The initial prodromes of type I BD are the signs and symptoms that occur before the first episode of mania (and the corresponding diagnosis), while relapse prodromes are the signs and symptoms that warn the patient that an episode of the disorder may be triggered [7]. The main reviews of prodromes in BD [7–16] (Supplementary Table S1) have drawn their evidence from studies that did not differentiate among children/adolescents, adults, and/or patients over 65 years of age. It is essential, therefore, to update knowledge of these signs and symptoms based on the recall and recognition of adults (18–65 years), as prodromes are not described or detected in the same way in different age groups. Children with BD may have difficulty expressing their experiences or ideas verbally [17], while elderly people with BD in the euthymic phase present substantial neuropsychological deficiencies [18] that may reduce their ability to detect cognitive changes prior to an episode of bipolar illness.

Expanding knowledge of the initial prodromes of BD will help to provide more information about its specificity and to test interventions before the onset of the disorder. Furthermore, increasing what is known about relapse prodromes will contribute to preventing and delaying the appearance of new episodes of BD using different pharmacological strategies (e.g., adjusting medication) and adjuvant psychological treatments.

Consequently, the primary objective of this systematic review is to determine the initial prodromes and relapse prodromes of manic, hypomanic, and depressive episodes that identify adult patients with type I and II BD. The secondary objectives are to determine their duration and classify the procedures used to detect them.

## Materials and Methods

The recommendations of Preferred Reporting Items for Systematic Reviews and Meta-Analyses: The PRISMA Statement [19] were followed to achieve the objectives of this review. The checklist was used to ascertain that all PRISMA recommendations were followed (Supplementary Table S2).

### Study selection criteria

The following inclusion criteria were used to select the studies: (1) research aimed at the detection of the initial prodromes and relapse prodromes of manic, hypomanic, and depressive episodes in patients diagnosed with type I or II BD; (2) publications in the English language; and (3) sample of adult patients (aged 18–65 years). The exclusion criteria were: (1) articles that did not identify BD prodromes; (2) review articles and meta-analyses; (3) single case studies; (4) studies with children, adolescents, and people aged over 65 years; (5) investigations that did not separate the results according to age group; (6) articles that included patients with diagnoses besides BD and did not separate the results based on such diagnoses; and (7) studies with patients who did not meet the DSM or International Classification of Diseases (ICD) criteria for a BD diagnosis.

### Search strategy

The databases search included PubMed, PsycINFO, and Web of Science from inception to January 2, 2018. The search strategy used in each of these databases was as follows: [“bipolar disorder” OR “manic-depressive illness”] AND [“symptoms” OR “phenomena”] AND [“initial” OR “early” OR “relapse” OR “prodrome” OR “premorbidity/premorbid” OR “prediction/predictors” OR “antecedents” OR “precursors” OR “early identification” OR “early recognition”]. Filters were used in the three databases to meet the inclusion criteria.

### Study selection process

In the article identification phase, the results of the three database searches were unified, and duplicate articles were eliminated. In the screening phase, the titles and abstracts of the articles that potentially fulfilled the inclusion criteria were read. This process was carried out by two reviewers independently (N.A.-G. and L.A.-C.) who then shared the results. Disagreements were resolved through a reasoned discussion between these two reviewers. When there was no agreement, the article in question was entirely reviewed. The eligibility phase consisted of reviewing and reading the full text of the articles preselected in the previous phase and the articles in question. This process was carried out by two reviewers independently (N.A.-G. and L.A.-C.), who later shared their findings; disagreements were resolved through a reasoned discussion between these two reviewers. When there was no agreement, a third expert reviewer (G.L.) decided whether the article met the inclusion criteria or not. Finally, in the inclusion phase, the articles were definitively selected for the present systematic review and prepared for extraction of relevant data.

### Process for data extraction from each study

Two reviewers (N.A.-G. and L.A.-C.) independently analyzed the selected articles. To facilitate and unify data extraction from each article, the reviewers used a template with six sections: title of the study, author/s and year of publication, sample size, characteristics of the participants, characteristics and quality of the study, and results (presence of initial and relapse prodromes of manic, hypomanic, and depressive episodes depending on the type of BD; prodrome duration; procedure for prodrome identification; and separation of results by age or type of diagnosis in studies with a nonadult population or those with a diagnosis other than BD). In cases in which the same prodrome received different designations, the two reviewers agreed on a term that would ensure the conceptual equivalence of such designations. Disagreements about data extracted from the selected articles were resolved by a third expert reviewer (G.L.).

Two authors (N.A.-G. and G.L.) independently assessed the risk of bias in the selected studies. Quantitative studies were assessed using a modified version of the Newcastle-Ottawa Scale [20] adapted for this systematic review (Supplementary Table S3). This version assessed the representativeness and sample sizes of the study groups, the comparison between participants and nonparticipants, the prodromes assessment tools, and the quality of the descriptive statistics. The quantitative selected studies were judged to be at low risk of bias (≥3 points) or high risk of bias (<3 points). Qualitative studies were evaluated using the Critical Appraisals Skills Programme checklist (CASP) for qualitative studies [21].

## Results

**Figure 1.**
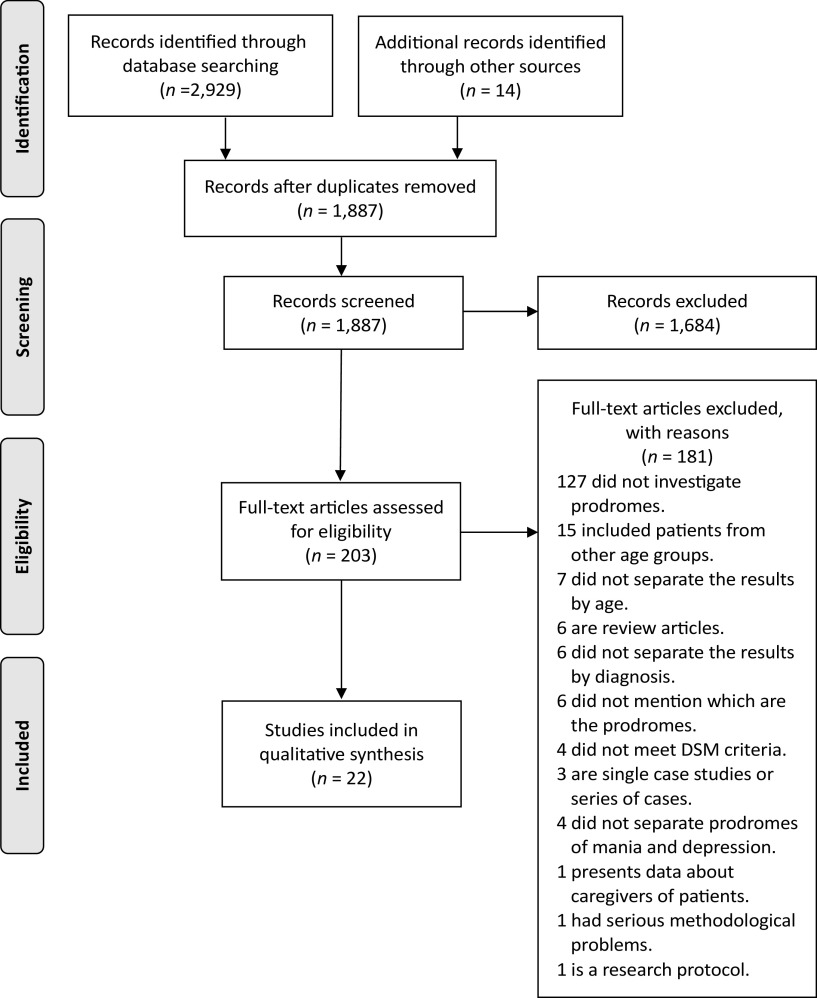
PRISMA diagram that illustrates the article selection process.

Twenty-two original studies that met the inclusion criteria were selected [4,22–42]. The selection process of these works is described in Figure 1. The main characteristics of the 22 studies are presented in Table 1. These works included a total of 1,809 adult patients with BD who experienced initial and relapse prodromes; according to the available data (*k =* 18), the weighted average age was 40.64 years. There were 754 patients who experienced initial prodromes (*k =* 6); in 4 of these 6 studies, the weighted average age of patients was 34.73 years (*n =* 135). There were 1,055 patients who experienced relapse prodromes (*k =* 16); in 14 of these 16 studies, the weighted average age of patients was 41.47 years (*n =* 965). In 21 studies, 38.62% of patients were male. Five studies incorporated data from the patients’ significant others. Two studies used a qualitative methodology. The results of the bias risk assessment of the 22 articles included in the study are in Supplementary Table S4.

**Table 1.** Characteristics of selected studies.

First author	Country	*N*	Average age (*SD*)	Male (%)	BD I (%)	No. of S-O^a^	Design	Quantitative methodology	Measurement of prodromes
Initial prodromes									
Benti et al. [22]	Australia	19	n/a	16.00	^b^	–	R	No	Ad hoc semi-structured interview and ad hoc self-report questionnaire
Estey et al. [4]	USA	30	29.60 (9.70)	26.67	30.00	30	R	Yes	Bipolar Scale of the CPNI-R
Hirschfeld et al. [33]	USA	600	n/a	34.00	^b^	–	R	Yes	Ad hoc self-report survey
Noto et al. [36]	Brazil	43	33.70 (6.80)	25.60	74.40	–	R	Yes	BPSS-R
Özgürdal et al. [37]	Germany	20	43.85 (9.38)	35.00	100	–	R	Yes^c^	Ad hoc semi-structured interview for mood swings
Zeschel et al. [38]	Germany	42	35.10 (10.00)	40.50	64.30	–	R	Yes	BPSS-R and ad hoc semi-structured interview for mood swings
Relapse prodromes									
Altman et al. [39]	USA	19	24.00 (3.40)	57.89	100	–	P	Yes	BPRS (expanded and shortened version)
Bauer et al. [40]	Germany and USA	59	n/a	33.90	62.71	–	P	Yes	ChronoRecord
Fletcher et al. [41]	Australia	13	40.50 (11.90)	46.20	0	–	R	No^d^	Ad hoc semi-structured interview
Goossens et al. [42]	Netherlands	111	47.23 (12.06)	35.00	67.00	–	R	Yes	Two questions^e^
Houston et al. [23]	USA	31	n/a	n/a	^b^	–	P	Yes	YMRS
Keitner et al. [24]	USA	74	42.00 (12.00)	47.00	100	45	R	Yes	Ad hoc open-ended self-report ^f^
Lam et al. [25]	United Kingdom	40	43.70 (13.10)	42.50	100	–	P	Yes	CPSI
Lobban et al. [26]	United Kingdom	96^g^	44.00 (10.40)	32.00	98.00	–	R	Yes	EWS checklists for mania and depression
Mander [27]	Australia	8	54.60 (10.80)	50.00	^b^	n/a	P	Yes	Semi-structured interview
Mantere et al. [28]	Finland	191^h^	37.70 (12.10)	47.10	47.10	–	R	Yes	Unstructured interview
Molnar et al. [29]	USA	20	37.65 (11.93)	45.00	^b^	6	R	Yes	Clinical interview
Perlman et al. [30]	USA	54	43.72 (11.46)	46.00	100	–	P	Yes	The sleep duration subscale of the PSQI
Ryu et al. [31]	South Korea	41	36.29 (12.06)	46.34	100	–	R	Yes	40-Item symptom checklist
		42	36.10 (9.54)	45.24	100	–			
Sahoo et al. [32]	India	30	33.80 (9.10)	70.00	100	30	R	Yes	Ad hoc scale of 83 items and unstructured interview
Smith and Tarrier [34]	Australia	20	43.90 (15.90)	45.00	^b^	–	R	Yes	40-Item symptom checklist plus additional questions^i^
Wong and Lam [35]	United Kingdom	206	44.00 (11.00)	40.00	^b^	–	R	Yes	One open-ended question

Abbreviations: *N*, total number of patients with bipolar disorder (BD) included in the study; *SD*, standard deviation; R, retrospective; CPNI-R, Coolidge Personality and Neuropsychological Inventory; BPSS-R, Bipolar Prodrome Symptom Scale-Retrospective; P, prospective; BPRS, Brief Psychiatric Rating Scale; YMRS, Young Mania Rating Scale; CPSI, The Coping with Prodromal Symptoms Interview; PSQI, Pittsburgh Sleep Quality Index.

a Number of significant-others of the patients.

b The study does not distinguish between BD I and BD II.

c The study also provides qualitative data.

d The study also provides quantitative data.

e “How can you tell if an episode of mania or depression is impending?” and “What is the first sign or behavior that you recognize in yourself that leads up to a manic or depressive episode?”

f “Please describe the behaviors you have experienced leading up to a manic or depressive episode. How can you tell that an episode is coming on?”

g Ninety-three patients completed the EWS (early warning signs) mania checklist and 89 patients completed the EWS depression checklist.

h Nineteen patients were excluded from the data analysis.

i The additional questions were about symptoms not included in the checklist.

### Initial and relapse prodromes

The percentages of initial prodromes extracted from four studies are shown in Table 2. To these are added the prodromes identified in two more works [4,22]. These two studies neither provided results for BD types nor distinguished between prodromes of manic/hypomanic and depressive episodes. In the qualitative study of Benti et al. [22], patients with BD stated that before being diagnosed, they needed increased hours of sleep and that they experienced anger, specific perseverant behaviors, and “up and down” moods. In the study by Zeschel et al. [38], 83.3% of the patients who participated also indicated that they had experienced mood swings before their first bipolar affective episode. Finally, Estey et al. [4] examined the symptoms experienced before 16 years of age in 30 adult patients with BD and compared them with 30 healthy adults. They also investigated the symptoms perceived by 30 significant others of the patients and compared them with 30 significant others of the healthy controls. Estey et al. [4] found that the 13 highest average scores among the patients were on the following items of the Bipolar Scale of the Coolidge Personality and Neuropsychological Inventory: easily bored, too hard on self, easily distracted, low self-esteem, easily regretful/guilty, fidgety, mood changed quickly, worried too much, easily irritated, temporary loss of interest, revengeful, depressed, and trouble with organization. These scores were significantly higher than those of healthy controls [4].

**Table 2.** Percentages of initial prodromes in patients with type I and II BD.

	BD I		BD II		BD I and II^a^	
Name of the prodrome	Mania	Depression	Hypomania	Depression	Mania/hypomania	Depression
Overly talkative	53.1%^b^	3.1%^b^	27.3%^b^		46.5–76.2%^b^^,^^c^^,^^d^	3.7–4.8%^b^^,^^d^
Racing thoughts^e^	46.9%^b^	6.3%^b^	36.4%^b^		44.2–75.0%^b^^,^^c^ ^,^^d^	4.8–7.4%^b^^,^^d^
Irritability^f^	43.8%^b^	28.1%^b^	36.4%^b^	45.5%^b^	41.9–67.0%^b^^,^^c^^,^^d^	19.0–51.8%^b^^,^^d^
Mood elevation^g^	37.5%^b^		18.2%^b^	9.1%^b^	32.6–77.0%^b^^,^^c^^,^^d^	3.7%^b^
Mood lability/Mood swings	33.3–56.3%^b^^,^^h^	9.4–66.6%^b^^,^^h^	36.4%^b^	27.3%^b^	26.2–51.2%^b^^,^^d^	22.2–31.0%^b^^,^^d^
Reckless or dangerous behavior	9.4%^b^	3.1%^b^			7.0–57.0%^b^^,^^c^^,^^d^	2.4–3.7%^b^^,^^d^
Impatience	53.1%^b^	18.8%^b^	36.4%^b^	9.1%^b^	48.8–57.1%^b^ ^,^^d^	21.4–25.9%^b^ ^,^^d^
Insomnia^i^	53.1%^b^	15.6%^b^	36.4%^b^		11.6–54.8%^b^ ^,^^d^	22.7–82.0%^b^ ^,^^c^ ^,^^d^
Physically agitated	50.0%^b^	15.6%^b^	27.3%^b^		44.2–78.6%^b^ ^,^^d^	
Depressed mood	46.9%^b^	28.1%^b^	54.5%^b^	36.4%^b^	2.4–48.8%^b^ ^,^^d^	48.1–83.0%^b^ ^,^^c^ ^,^^d^
Increased energy or goal-directed activity	46.9%^b^		18.2%^b^		39.5–73.0%^b^ ^,^^c^	
Anxiety	43.8%^b^	25.0%^b^	45.5%^b^	27.3%^b^	31.0–44.2%^b^ ^,^^d^	40.7–64.3%^b^ ^,^^d^
Tiredness or lack of energy	37.5%^b^	28.1%^b^	27.3%^b^	45.5%^b^	9.5–34.9%^b^ ^,^^d^	51.8–76.2%^b^ ^,^^c^ ^,^^d^
Social isolation	28.1%^b^	25.0%^b^		36.4%^b^	7.1–25.3%^b^ ^,^^d^	44.4–69.0%^b^ ^,^^d^
Decreased school or work functioning	25.0%^b^	18.8%^b^	18.2%^b^	9.1%^b^	14.3–23.3%^b^ ^,^^d^	25.9–69.0%^b^ ^,^^c^ ^,^^d^
Decreased need for sleep	25.0%^b^	3.1%^b^	27.3%^b^	9.1%^b^	71.4%^d^	
Overly self-confident	21.9%^b^		18.2%^b^	9.1%^b^	20.9–69.0%^b^ ^,^^d^	2.4–3.7%^b^ ^,^^d^
Feeling worthless or guilty	21.9%^b^	6.3%^b^	18.2%^b^	36.4%^b^	4.8–20.9%^b^ ^,^^d^	22.2–79.0%^b^ ^,^^c^ ^,^^d^
Suspiciousness	21.9%^b^	9.4%^b^	18.2%^b^	9.1%^b^	16.7–20.9%^b^ ^,^^d^	14.8–23.8%^b^ ^,^^d^
Weight loss or decrease in appetite	18.8%^b^	3.1%^b^	9.1%^b^	9.1%^b^	20.9–28.6%^b^ ^,^^d^	7.4–40.5%^b^ ^,^^d^
Difficulty making decisions	18.8%^b^	6.3%^b^	36.4%^b^	27.3%^b^	11.9–23.3%^b^ ^,^^d^	18.5–61.9%^b^ ^,^^d^
Inversion of the sleep/wakefulness pattern	18.8%^b^	6.3%^b^			14.0–78.0%^b^ ^,^^c^	7.4%^b^
Overly creative	15.6%^b^		18.2%^b^	9.1%^b^	16.3–54.8%^b^ ^,^^d^	3.7–9.5%^b^ ^,^^d^
Defiant behavior	15.6%^b^	3.1%^b^	9.1%^b^	9.1%^b^	14.0–35.7%^b^ ^,^^d^	2.4–7.4%^b^ ^,^^d^
Thinking about suicide	12.5%^b^	12.5%^b^	9.1%^b^		7.1–11.6%^b^ ^,^^d^	14.8–70.0%^b^ ^,^^c^ ^,^^d^
Obsessions or compulsions^j^	12.5%^b^		9.1%^b^		2.4–11.6%^b^ ^,^^d^	7.1%^d^
Increased sexual energy	12.5%^b^		9.1%^b^		11.6–31.0%^b^ ^,^^d^	2.4%^d^
Weight gain or increase in appetite	9.4%^b^	9.4%^b^	9.1%^b^	18.2%^b^	4.8–34.9%^b^ ^,^^d^	21.4–22.7%^b^ ^,^^d^
Hypersomnia	6.3%^b^	12.5%^b^	9.1%^b^	18.2%^b^	7.0–7.1%^b^ ^,^^d^	22.7–33.3%^b^ ^,^^d^
Hallucinatory experiences	6.3%^b^	6.3%^b^			4.7–4.8%^b^ ^,^^d^	
Self-harming behavior	3.1%^b^				2.3–4.8%^b^ ^,^^d^	4.8%^d^
Risky sexual behavior	3.1%^b^				2.3–19.0%^b^ ^,^^d^	
Strange or unusual ideas			9.1%^b^		2.3–19.0%^b^ ^,^^d^	4.8%^d^
Decreased concentration	37.5%^b^	12.5%^b^	36.4%^b^	36.4%^b^	37.2%^b^	29.6%^b^
Overly cheerful	37.5%^b^	3.1%^b^	9.1%^b^	9.1%^b^	30.2%^b^	7.4%^b^
Anhedonia	31.3%^b^	9.4%^b^	36.4%^b^	27.3%^b^	44.2%^b^	22.2–72.0%^b^ ^,^^c^
Difficulty thinking or communicating clearly	18.8%^b^	12.5%^b^	9.1%^b^	9.1%^b^	16.3%^b^	18.5%^b^
Physically slowed down	9.4%^b^	18.8%^b^	18.2%^b^	27.3%^b^	11.6%^b^	33.3%^b^
Extremely energetic/active					85.7%^d^	4.8%^d^
Poor judgment					72.0%^c^	
Disturbed diurnal rhythm					47.6%^d^	31.0%^d^
Lack of concentration					35.7%^d^	59.5–76.0%^c^ ^,^^d^
Thought disorder					26.2%^d^	45.2%^d^
Immature behavior					26.2%^d^	2.4%^d^
Physical exhaustion					11.9%^d^	78.6%^d^
Reduce vitality						81.0%^d^
Suicide attempts						2.4%^d^

a The studies did not distinguish prodromes by type of BD.

b Noto et al. [36].

c Hirschfeld et al. [33].

d Zeschel et al. [38].

e Racing thoughts/increased speech in Hirschfeld et al. [33].

f Excessive irritability/aggressive behavior in Hirschfeld et al. [33].

g Heightened mood/elation in Hirschfeld et al. [33].

h Özgürdal et al. [37].

i Insomnia/excessive sleep in Hirschfeld et al. [33].

j Only compulsions in Zeschel et al. [38].

The percentages of relapse prodromes extracted from 11 studies are presented in Table 3. To these are added the prodromes identified in five more works. First, Perlman et al. [30] found that a shorter duration of sleep predicted a greater severity of depressive episodes across a 6-month follow-up. However, Bauer et al. [40] found that a decrease in patients’ sleep hours or bedrest hours was followed by a shift to hypomania/mania on the next day and that an increase in sleep or bedrest was followed by a change to depression. Goossens et al. [42] found that, among the categories of mania prodromes they used, 21% of patients experienced an increase in energy level; 17% a decreased need for sleep; 16% an increase in social functioning; 9% cognitive activity characterized by, among other prodromes, racing thoughts and feeling anxious; 4% problems with impulse control; and 3% not able to concentrate. Among the categories of depression prodromes they used, 15% of patients experienced cognitive activity that included, among other prodromes, feeling anxious, negative thoughts, and feeling guilty, 12% had depressed mood and a lower level of energy, 11% a reduction in social functioning, 10% sleep problems, 5% not able to concentrate, 3% problems related to impulse control, and 2% lowered self-esteem and weight loss or poor appetite. Regarding the information provided by patients and relatives, Keitner et al. [24] found that, among the mania prodrome categories, 35% of patients experienced cognitive symptoms (e.g., cannot concentrate) that were identified by 18% of relatives; 22% had behavioral and neurovegetative symptoms (e.g., more talkative, decreased sleep, and more energy) reported by 47% and 13% of relatives, respectively; and 15% had mood symptoms (e.g., feeling high and irritability) detected by 17% of relatives. Among the prodromes of depression, 31% of patients experienced cognitive symptoms (e.g., poor concentration) identified by 33% of relatives; 23% had neurovegetative symptoms (e.g., loss of appetite and energy) reported by 27% of relatives; 20% had mood symptoms (e.g., crying and irritable) detected by 18% of relatives; 10% had behavioral symptoms (e.g., quiet) indicated by 13% of relatives; and 7% experienced a reduction in social functioning (e.g., withdrawal from friends), revealed by 5% of family members [24]. Finally, Sahoo et al. [32], after combining the information of patients and relatives, found that the most frequent mania prodromes were hostility, overactivity, ideas of grandiosity, meddling and arguing, reduced sleep, not needing much sleep, irritability, elation, pressure of speech, overspending, distractibility, being uncooperative, senses seem sharper, increased self-care, less affectionate, less responsible, ideas of persecution, concentration difficulty, labile emotional experience, involved in many projects, and increased sexual interest. Regarding idiosyncratic relapse prodromes, in the work of Smith and Tarrier [34], more than 70% of patients identified diverse symptoms such as: “getting very angry with my ex-wife,” “increased sensitivity to racism,” and “cutting her own face.” Furthermore, in Sahoo et al. [32], 47% of participants (patients and family members) mentioned the following mania prodromes: increased religiosity, making decisions easily, reddening of eyes, being abusive, listening to loud music, recalling past events, and ideas of reference. Lastly, the idiosyncratic relapse prodromes identified in the study of Wong and Lam [35] are in Table 3.

**Table 3.** Percentages of relapse prodromes in patients with type I and II BD.

	BD I		BD II		BD I and II^a^	
Name of the prodrome	Mania	Depression	Hypomania	Depression	Mania/hypomania	Depression
Sleep disturbances/decreased need for sleep	13.8–85.7%^b^ ^,^^c^ ^,^^d^		11.8–72.7%^c^ ^,^^e^		10.5–90.0%^f^ ^,^^g^ ^,^^h^ ^,^^i^ ^,^^j^ ^,^^k^	6.0–57.1%^i^ ^,^^j^
Elevated mood	10.5–87.8%^b^ ^,^^d^		5.9%^c^		12.5–100.0%^f^ ^,^^g^ ^,^^h^ ^,^^i^ ^,^^j^ ^,^^k^	6.0%^j^
More talkative than usual	3.4–78.6%^c^ ^,^^d^		5.9–72.7%^c^ ^,^^e^		10.5–93.0%^f^ ^,^^g^ ^,^^h^ ^,^^i^ ^,^^j^ ^,^^k^	
Increased energy/More goal-directed behavior	44.7–80.5%^b^ ^,^^d^		81.8%^e^		22.0–93.0%^g^ ^,^^h^ ^,^^j^ ^,^^k^	
Irritable	6.9–63.4%^b^ ^,^^c^ ^,^^d^	2.6%^c^		5.2%^c^	25.0–60.0%^f^ ^,^^g^ ^,^^i^ ^,^^j^ ^,^^k^	28.6–47.0%^g^ ^,^^i^ ^,^^j^
Thoughts start to race	15.8–73.8%^b^ ^,^^d^		5.9%^c^		29.0–93.0%^g^ ^,^^i^ ^,^^j^ ^,^^k^	
Increased self-esteem^l^	6.9–63.4%^b^ ^,^^c^ ^,^^d^		5.9%^c^		16.0–93.0%^g^ ^,^^h^ ^,^^i^ ^,^^j^ ^,^^k^	
Difficulty concentrating	52.4–53.7%^d^				12.5–67.0%^g^ ^,^^h^ ^,^^i^ ^,^^j^	62.0–82.0%^g^ ^,^^i^ ^,^^j^
Feeling anxious/Restlessness	10.5–53.7%^b^ ^,^^d^	5.3–18.4%^b^ ^,^^c^		6.9–60.0%^c^ ^,^^e^	7.0–49.0%^g^ ^,^^j^ ^,^^k^	53.0–82.0%^g^ ^,^^j^
Strong interest in sex	14.3–24.4%^d^				10.5–87.0%^f^ ^,^^g^ ^,^^i^ ^,^^j^ ^,^^k^	
Increase in activity^m^	3.4%^c^				21.1–100.0%^f^ ^,^^g^ ^,^^h^ ^,^^i^ ^,^^k^	
Spending too much	13.2–68.3%^b^ ^,^^d^				19.0–80.0%^g^ ^,^^j^ ^,^^k^	
Unusual thought content	26.8–80.0%^d^ ^,^^n^	17.0%^n^			18.0–67.0%^g^ ^,^^j^ ^,^^k^	
Somatic symptoms^o^	3.4–9.8%^c^ ^,^^d^				13.0–20.0%^i^ ^,^^j^	20.0–35.7%^g^ ^,^^i^ ^,^^j^
Being uninhibited or outrageous	40.5–56.1%^d^				15.0–67.0%^g^ ^,^^j^ ^,^^k^	
Weight loss or poor appetite	5.3–43.9%^b^ ^,^^d^	7.9–13.2%^b^			13.0–33.0%^j^ ^,^^k^	18.0–53.0%^g^ ^,^^i^ ^,^^j^
Feeling very religious	23.8–31.7%^d^				13.0–73.0%^g^ ^,^^j^ ^,^^k^	6.0–12.0%^j^
Senses seem sharper^p^	50.0–63.4%^d^				25.0–93.0%^g^ ^,^^j^	
Insomnia	54.8–58.5%^d^	5.3–26.3%^b^ ^,^^c^		3.4%^c^	67.0–80.0%^g^ ^,^^j^	18.0–51.0%^g^ ^,^^j^
Feeling creative	40.5–53.7%^d^				57.0–93.0%^g^ ^,^^j^	
Feeling strong or powerful	42.9–46.3%^d^				30.0–80.0%^g^ ^,^^j^	
Feeling in another world	31.0–46.3%^d^				22.0–73.0%^g^ ^,^^j^	6.0–12.0%^j^
Hearing hallucination	19.5–45.2%^d^				12.0–27.0%^g^ ^,^^j^	
Involved in many projects	21.4–39.0%^d^				32.0–73.0%^g^ ^,^^j^	
Being uncooperative	14.3–19.5%^d^				27.0–47.0%^g^ ^,^^j^	21.0–41.0%^g^ ^,^^j^
Visual hallucination	7.1–9.8%^d^				7.0–27.0%^g^ ^,^^j^	
Thinking my thoughts are controlled	7.3–9.5%^d^				16.0–40.0%^g^ ^,^^j^	6.0–12.0%^j^
Negative thinking/Worrying a lot	26.8–28.6%^d^	7.9–13.2%^b^		100.0%^e^	7.0–13.0%^h^ ^,^^j^	57.0–82.0%^g^ ^,^^j^
Neglect hygiene and appearance	4.8–14.6%^d^				5.3–13.0%^f^ ^,^^j^	35.0–53.0%^g^ ^,^^j^
Depressed mood	35.7–48.8%^d^	5.3–15.8%^b^		8.6%^c^	7.0–27.0%^j^	42.9–88.0%^g^ ^,^^i^ ^,^^j^
Low in energy-tired	26.2–43.9%^d^	7.9–26.3%^b^ ^,^^c^		17.2–60.0%^c^ ^,^^e^	7.0%^j^	71.0–100.0%^g^ ^,^^i^ ^,^^j^
Afraid of going crazy	9.8–31.0%^d^				20.0–40.0%^j^	18.0–35.0%^g^ ^,^^j^
Nothing seems enjoyable	19.5–26.2%^d^				7.0%^j^	82.0–94.0%^j^
Cannot face normal task	23.8–24.4%^d^				7.0%^j^	62.0–94.0%^g^ ^,^^j^
Thinking about death or suicide	14.3–22.0%^d^				7.0%^j^	20.0–65.0%^g^ ^,^^i^ ^,^^j^
Low interest in sex	14.6–21.4%^d^				7.0%^j^	51.0–82.0%^g^ ^,^^j^
Do not feel like seeing people	16.7–19.5%^d^				7.0–13.0%^j^	56.0–100.0%^g^ ^,^^j^
Feeling very guilty	9.5–14.6%^d^	2.6%^c^			7.0%^j^	44.0–77.0%^g^ ^,^^j^
Cannot get up in the morning	9.5–9.8%^d^	13.2%^b^		70.0%^e^	7.0–13.0%^j^	48.0–94.0%^g^ ^,^^j^
Low in self-confidence	9.5–9.8%^d^				7.0%^j^	88.0–100.0%^j^
Alcohol abuse					23.0–35.0%^g^ ^,^^i^	14.3–20.0%^g^ ^,^^i^
Reckless pleasure-seeking^q^					8.0–17.0%^g^ ^,^^i^	6.0%^g^
Conceptual disorganization	17.0%^n^	83.0%^n^			5.3–16.7%^f^	
Increased sociability	18.4–21.1%^b^					
Psychomotor agitation	3.4%^c^		5.9%^c^	1.7%^c^		28.6–45.0%^g^ ^,^^i^
Harmful activities	3.4%^c^					
Improved memory					12.5%^h^	
More benevolent					12.5%^h^	
Lack of insight					8.3–10.5%^f^	
Disruptive-aggressive behavior					5.3–8.3%^f^	
Loss of interest^r^		10.5–36.8%^b^ ^,^^c^		5.2%^c^		57.1–63.0%^g^ ^,^^i^
Low motivation		7.9–10.5%^b^				75.0%^g^
Indecisiveness		2.6%^c^		1.7%^c^		
Hypersomnia		2.6%^c^				28.6–42.0%^g^ ^,^^i^
Feeling physically slowed				60.0%^e^		
Diminished ability to think				1.7%^c^		51.0%^g^
Less talkative						58.0%^g^
Senses seem duller						46.0%^g^
Using sleeping tablets						17.0%^g^

a The studies did not distinguish prodromes by type of BD.

b Lam et al. [25].

c Mantere et al. [28].

d Ryu et al. [31].

e Fletcher et al. [41].

f Houston et al. [23].

g Lobban et al. [26].

h Mander [27].

i Molnar et al. [29].

j Smith and Tarrier [34].

k Wong and Lam [35].

l Grandiose plans in Wong and Lam [35].

m Wanting to party all night in Lobban et al. [26].

n Altman et al. [39].

o Lots of aches and pains in Ryu et al. [31] and Smith and Tarrier [34].

p Colours brighter/more vivid in Lobban et al. [26].

q Taking street drugs in Lobban et al. [26].

r Loss of interest in activity or people in Lam et al. [25].

### Duration of prodromes

Two articles reported about the duration of the initial prodromes. The average length of the prodromal phase of mania/hypomania was 1.5 months in Zeschel et al. [38], and 33.26 and 43.54 months for prodromes of mania and hypomania, respectively, in Noto et al. [36]. Additionally, the prodromal phase of depression lasted 4.3 months in Zeschel et al. [38], and 9.88 months in patients with BD I and 31.87 months in BD II, in the study by Noto et al. [36].

Seven articles included data about the duration of relapse prodromes [27–29,31,32,34,39]. The average duration of the mania/hypomania prodromes fluctuated between 7.0 and 30.0 days [27,29,32,34,39]. On the other hand, the average duration of the prodromes of depression fluctuated between 10.9 and 120.0 days [29,34,39]. Additionally, Mantere et al. [28] found that the median of the prodromal phase of mania was 17.0 days (12.0 days in hypomania) and that the median of the prodromal phase of depression was 31.0 days. Finally, Ryu et al. [31] found that the average duration of manic prodromes was 4.5 weeks (in patients without psychotic symptoms in the most recent episode) and 6.9 weeks (in patients with psychotic symptoms).

### Prodrome identification procedures

Four of the six selected articles that studied initial prodromes used semi-structured interviews to identify them [22,36–38]; three of the four semi-structured interviews were developed ad hoc [22,37,38]; two of the four used the Bipolar Prodrome Symptom Scale-Retrospective [36,38]. Benti et al. [22] also used a questionnaire prepared ad hoc. The remaining two investigations used a self-administered survey [33] and a self-report inventory [4].

Ten of the 16 articles that studied relapse prodromes used different tools in their clinical interviews [23,25,27–29,31,32,34,39,41]. Altman et al. [39] and Houston et al. [23] interviewed patients using the Brief Psychiatric Rating Scale and the Young Mania Rating Scale, respectively; Ryu et al. [31] and Smith and Tarrier [34] employed a 40-item symptom checklist to interview patients. Sahoo et al. [32] also used a symptom checklist, in which patients selected their relapse prodromes among different items. The remaining six studies used a symptom checklist that was mailed to patients [26]; some open-ended questions [24,35,42]; ChronoRecord software [40]; and the sleep duration subscale of the Pittsburgh Sleep Quality Index [30].

## Discussion

The primary objective of this systematic review was to determine the initial and relapse prodromes of manic, hypomanic, and depressive episodes that identify adult patients with type I and II BD. The prodromes cited most in the selected works are shown in Table 4. The duration of the initial prodromal phases of mania/hypomania and depression was greater than the duration of the relapse prodromal phases of these episodes. Additionally, the method used most to identify both types of prodromes was the clinical interview.

**Table 4.** Prodromes most cited in the literature that are identified by adult patients with BD.^a^

Initial prodromes		Relapse prodromes	
Manic/hypomanic episodes	Depressive episodes	Manic/hypomanic episodes	Depressive episodes
Overly talkative (*k =* 3)	Mood lability/mood swings (*k =* 3)	Sleep disturbances/decreased need for sleep (*k =* 13)	Low in energy-tired (*k =* 8)
Racing thoughts (*k =* 3)	Insomnia (*k =* 3)	Elevated mood (*k =* 10)	Feeling anxious/restlessness (*k =* 6)
Irritability (*k =* 3)	Depressed mood (*k =* 3)	More talkative than usual (*k =* 10)	Weight loss or poor appetite (*k =* 6)
Mood elevation (*k =* 3)	Tiredness or lack of energy (*k =* 3)	Increased energy/more goal-directed behavior (*k =* 9)	Depressed mood (*k =* 6)
Mood lability/mood swings (*k =* 3)	Decreased school or work functioning (*k =* 3)	Irritable (*k =* 9)	Irritable (*k =* 5)
Reckless or dangerous behavior (*k =* 3)	Feeling worthless or guilty (*k =* 3)	Thoughts start to race (*k =* 8)	Difficulty concentrating (*k =* 5*)*
	Thinking about suicide (*k =* 3)	Increased self-esteem (*k =* 8)	Insomnia (*k =* 5)
		Difficulty concentrating (*k = 7*)	Negative thinking/worrying a lot (*k =* 5)
		Feeling anxious/restlessness (*k =* 6)	Feeling very guilty (*k =* 4)
		Strong interest in sex (*k =* 6)	Loss of interest (*k =* 4)
		Increase in activity (*k =* 6)	Hypersomnia (*k =* 4)
		Spending too much (*k =* 5)	
		Unusual thought content (*k =* 5)	

*k =* number of studies.

a The following relapse prodromes of mania: overactivity, does not need much sleep, irritability, elation, and concentration difficulty, identified by Sahoo et al. [32] have not been included in this table since they were derived from a combination of patients’ and relatives’ information.

### Initial and relapse prodromes

Previous reviews of initial and relapse prodromes in patients with BD are heterogeneous (Supplementary Table S1). Despite this, the most frequent initial prodromes of mania and depression identified in this review (Table 4) were also detected in one or more of the reviews carried out by Conus et al. [7], Howes et al. [9], Leopold et al. [12], Malhi et al. [13], Skjelstad et al. [15], and Van Meter et al. [16]. Likewise, the relapse prodromes of episodes of mania and depression more frequently found in this review (Table 4) were also identified in one or more of the reviews carried out by Fava [8], Jackson et al. [10], Lam and Wong [11], Sierra et al. [14], and Van Meter et al. [16].

A variable number of adult patients identified initial and relapse prodromes. In three studies, for example, between 6.3% and 79.0% of patients identified feeling worthless or guilty as an initial prodrome of depressive episodes; this was produced by the interindividual variability of patients when they are experiencing prodromes [43] and by the heterogeneity of the procedures used to identify them. Interindividual variability depends, among other factors, on the stage of the patient’s disorder, the presence or absence of psychotic symptoms in the most recent episode of the bipolar illness [31], patient temperament type [44], and the existence or not of comorbidity. Additionally, different types of methods with different response options were used to identify the prodromes. Furthermore, the different interviews varied in their degree of structuring and questions asked of the patients (some interviews were elaborated ad hoc).

Patients detected 5 initial and 29 relapse prodromes before the same type of episode of BD (Tables 2 and 3). This result shows the low specificity of the initial prodromes, which is consistent with the reviews of Conus et al. [7] and Skjelstad et al. [15] that included patients of different ages. Among the most frequent prodromes (Table 4) only the relapse prodromes of more talkative than usual, increased energy/more goal-directed behavior, thoughts start to race, increased self-esteem, strong interest in sex, increase in activity, and spending too much were identified exclusively before a manic/hypomanic episode, while loss of interest and hypersomnia were detected only before a depressive episode. Although these relapse prodromes seem to be promising in predicting new episodes of BD, the number of studies from which they were derived was very limited in some cases.

The initial prodrome called mood lability/mood swings warrants particular attention. This prodrome was identified in three studies before a manic/hypomanic episode and a depressive episode [36–38]. Furthermore, the study by Benti et al. [22] identified “up and down” moods, and the study by Estey et al. [4] detected “mood changed quickly,” although both studies did not distinguish whether these prodromes preceded a manic/hypomanic or depressive episode. The initial prodrome called mood lability/mood swings has been identified by patients of different ages in the reviews of Conus et al. [7], Howes et al. [9], Leopold et al. [12], Malhi et al. [13], Skjelstad et al. [15], and Van Meter et al. [16]. According to Özgürdal et al. [37], mood swings occur before the bipolar illness without justified cause, and according to Benti et al. [22], they are not related to specific situations. The importance of this nonspecific initial prodrome is manifested in the work of Angst et al. [45], who found that “ups and downs of mood” are the strongest risk factor for BD, which, in their opinion, are produced by a lability and/or lack of maturation of the mood-regulating system.

Patients with types I and II BD show specific characteristics and appear to have varied responses to different mood stabilizers [46]. Only one of the studies reviewed here examined the differences between both types of BD regarding initial prodromes [36] and one regarding relapse prodromes [28]; thus, it was not possible to obtain more evidence related to the specificity of these prodromes in each type of BD.

The evidence on the agreement between the prodromes detected by patients and relatives is scant but promising. Among the studies selected in this review, Keitner et al. [24] and Molnar et al. [29] found a moderate degree of agreement between patients and family members regarding the identification of relapse prodromes of mania and depression. Furthermore, Sahoo et al. [32], not having found significant differences between patients and relatives regarding the number and type of relapse prodromes of mania, combined the information provided by patients and relatives.

The initial prodromes of mania/hypomania and depression identified in the majority of studies could be part of stages 1a (mild or nonspecific symptoms) and 1b (prodrome) of the potential clinical staging model of Berk et al. [47], highlighted years later by Singh et al. [48] and Berk et al. [49]. Taking into account the different risk factors of developing the disorder (stage 0) and the most frequent initial prodrome (although they have low specificity) will help clinicians to identify people who are at risk for BD. Currently, there is not enough evidence in favor of specific treatments in stage 1 [48]. Despite this, it is recommended to monitor a person’s mental state and encourage him/her to lead a healthy lifestyle, be knowledgeable about the symptoms of the disorder, and know when to ask for professional help [48]. Furthermore, the most frequent relapse prodromes can herald new episodes of the disorder, and these prodromes could be included in stages 3b and 3c. Recognition of relapse prodromes (in which the patient can be trained), and subsequent pharmacological and psychological management, will prevent and delay the appearance of new episodes, which is important given the recurrent nature of BD.

It is necessary to distinguish the prodromes identified in this review from subsyndromal symptomatology (SS) and mood instability (MI). The SS has a lower intensity than that required to diagnose an episode of BD [50]. However, subsyndromal depressive symptoms are associated with a significant functional impairment in patients with types I and II BD [51] and the term symptomatic density has been coined to refer to the percentage of time that a patient with BD experiences SS throughout his/her illness [50]. MI refers to the syndromic or subsyndromic fluctuations of energy and mood of patients with BD [50] that predict long-term functional outcomes [52]. Therefore, SS and MI dominate the clinical course of the patient with BD and are associated with the general functional impairments that represent an important part of the disease burden.

### Duration of prodromes

Initial prodromes lasted longer than relapse prodromes. This result is in line with Conus et al. [7], who indicated that people can experience minor symptoms of the disorder up to 10 years before the acute onset. The duration range of relapse prodromes of depressive episodes was greater than the duration range of relapse prodromes of mania/hypomania episodes (range 10.9–120.0 days vs. 7.0–48.3 days). This finding is consistent with the reviews of Jackson et al. [10], Lam and Wong [11], and Sierra et al. [14] that included patients of different ages. This difference may be due to prodromes of depression being more difficult to detect and encompassing a greater diversity of symptoms [11]. Nevertheless, these results should be taken with caution due to the small number of studies that measured the duration of the initial prodromes (*k* = 2), the small sample size of some studies, the lack of distinction between type I and II BD in some studies, the differences in procedures for identifying prodromes, the variability between patients regarding the duration of the prodromes (e.g., Molnar et al. [29]), the time elapsed between the presence of the prodrome and the moment of its identification, and the presence of comorbidity in patients.

### Prodrome identification procedures

The most used method to identify initial and relapse prodromes in the selected studies was the clinical interview. The interview elicits an immediate response from patients. Additionally, it allows clinicians to adapt their language to the educational level of patients, reformulate the questions if the case requires it, and observe patients’ nonverbal behavior. In an interview, however, a patient may tend to give less information on prodromes [15], and conducting them requires more personal resources and is more expensive [11]. Regarding symptoms checklists that are administered without an interview, patients may tend to indicate prodromes indiscriminately [11,15]; this can also occur with questionnaires and inventories. Nevertheless, these methods allow patients to think through their answers, are cheaper than interviews, are easy to apply, can be sent by mail (e.g., Benti et al. [22]; Lobban et al. [26]), and, if desired, the respondent’s anonymity can be guaranteed, giving them greater freedom to answer.

It is important to consider the use of digital technologies to identify BD prodromes. According to Monteith et al. [53], there are applications in which the patient plays an active role and answers questions about his/her illness (e.g., the ChronoRecord software of Bauer et al. [54]) and other applications that detect patients’ moods passively from their activity on the Internet and sensors installed on smartphones and clothes (see Grünerbl et al. [55]). It is proposed, therefore, that the most frequent initial and relapse prodromes found in this review be incorporated into active and passive monitoring systems. In particular, it is suggested that an app be developed for smartphones that has, among other components, an active system allowing the patient to identify the most frequent prodromes mentioned in the literature (Table 4) and record the idiosyncratic prodromes (and the factors that contribute to their appearance [e.g., toxic consumption, other diseases, etc.]). This app must also have a passive system that detects the most frequent behavioral prodromal symptoms through a GPS, an accelerometer, and a call recorder. Regarding initial prodromes, this technology would allow monitoring of the functioning of people at risk for BD. Moreover, this app would make it possible to identify the frequency, intensity, and duration of important relapse prodromes. Thus, this monitoring system would constitute a source of big data, allowing better knowledge of the signs and symptoms that precede the beginning of a BD episode in a given patient. If these data were coupled with information from relatives or close friends of the patient, the clinician would be in a better position to make effective and fast clinical decisions.

### Limitations

This review has the following limitations. To begin with, 72.7% of the selected studies used a retrospective design; therefore, it is possible that knowledge acquired by patients about BD influenced their recall of the identified prodromes [15]. Further, the sample size of 40.9% of the selected works was ≤30 patients, which limits generalization of the results. The heterogeneity of prodrome identification procedures resulted in some differences in the designation of the same prodromal symptoms. Despite this, the reviewers reached a strong consensus about terms that ensured the conceptual equivalence of different designations of the same prodrome. Furthermore, only one study (Lam et al. [25]) examined whether there were gender differences in the detection of prodromal symptoms. Finally, 90.9% of the selected studies did not include a comparison group, which resulted in lower specificity of the prodromes identified.

### Conclusions and future research directions

Previous reviews of prodromes of BD have examined temperament [7], affective lability [12], and comorbidity [12] before the initial episode. Other reviews have distinguished between the retrospective and prospective methodology used in the selected studies [9,12,13], have differentiated between distal and proximal initial prodromes [7,15], and have examined the sensitivity and specificity of the detected prodromes [9,15]. The current systematic review has paid attention to the initial prodrome called mood lability/mood swings, has estimated that more than 70% of the selected studies used a retrospective design, and has underlined the low specificity of the initial prodromes detected. Most importantly, based on the information provided by a well-defined population of patients, the results of this review provide a specific tool for clinicians and researchers: a list that contains the initial and relapses prodromes most commonly cited in the scientific literature (Table 4).

In conclusion, in the present review, adult patients with type I and II BD identified initial and relapse prodromes of manic, hypomanic, and depressive episodes. The most frequent initial prodromes have low specificity. The most frequent relapse prodromes, more talkative than usual, increased energy/more goal-directed behavior, thoughts start to race, increased self-esteem, strong interest in sex, increase in activity, and spending too much, appear to be effective in predicting new episodes of BD. Evidence about the correspondence between the prodromes detected by patients and relatives is scant but promising. Regarding duration, initial prodromes last longer than relapse prodromes. Furthermore, relapse prodromes of depressive episodes have a longer duration than relapse prodromes of episodes of mania/hypomania. Finally, the most used prodrome identification procedure is the clinical interview.

Future research about prodromes should: (1) compare the information of adult patients with BD with that of adults with other mental disorders and with healthy controls to establish specific initial prodromes; confirm the specificity of the most frequent relapse prodromes identified in this review; and determine that, indeed, they are specific prodromes of BD; (2) compare the prodromes detected by children/adolescents, adults, and elderly individuals (given the differences in describing and identifying them); (3) examine the neurobiological bases of the prodromal phases (as have been investigated in schizophrenia; see the review by Fusar-Poli et al. [56]); and (4) analyze the ability of different procedures to detect the prodromes of BD in the main age groups (e.g., paper and pencil questionnaires vs. digital technologies). To perform the above, it is necessary to use widely accepted designations of the prodromes, well validated and appropriate identification tools for the subject’s age, conduct functional neuroimaging studies comparing different groups of people, conduct multisite studies that improve the generalization of results [16], and conduct projects with digital technologies aimed at obtaining big data on BD prodromes.

Knowledge about the prodromes of BD and their predictive capacity is necessarily complemented with knowledge of risk factors (e.g., genetic factors, physical or sexual abuse in childhood), vulnerability factors (biological, behavioral, developmental, and personality factors), and pathophysiological changes that may be present in people at high risk of developing BD or experiencing a relapse. Increasing knowledge in these areas will allow establishment of high-risk profiles and testing specific interventions that help prevent the onset of the disorder and new episodes, and consequently, reduce patient suffering.

## Supplementary material

For supplementary material accompanying this paper visit https://doi.org/10.1192/j.eurpsy.2019.18. click here to view supplementary material
