# Supplementary material for: Initial and relapse prodromes in adult patients with episodes of bipolar disorder: A systematic review
Source: Eur Psychiatry. 2020 Feb 12;63(1):e12. doi: 10.1192/j.eurpsy.2019.18 (PMC7315869; doi:10.1192/j.eurpsy.2019.18)
Supplement: Supplementary file 1 [file S092493381900018Xsup.zip › S092493381900018Xsup001.docx]

| **Supplementary Table S1.** Characteristics of the main reviews about initial and relapse prodromes in patients with bipolar disorder | | | | | | | | |
| --- | --- | --- | --- | --- | --- | --- | --- | --- |
| First author | Type of prodrome identified | PRISMA rules | Databases consulted | *k* | Patient sample | Duration of prodromes | Prodrome identification procedure | Main prodromes identified |
| Conus et al [7] | Initial | No | Medline, PubMed, PsycLIT, and a hand search of relevant literature | 19^a^ | 969 subjects (children, adolescents, and adults with BD I, BD II, mania with psychotic features or MD with psychotic features) | n/a | Postal survey, medical histories, combination of open-ended and semi-structured questionnaires | Distal prodromes: depressed mood, hopelessness, mania/hyperactivity, mixed symptoms, mood swings/episodic mood change, elevated mood, reduction of sleep/decreased need for sleep, functional impairment, anger/anger dyscontrol, irritability, changed energy level, and conduct problems.  Proximal prodromes: elevated mood, mood swings, sleep disturbances/disturbed sleep, anger/irritability, increased energy/activity, anxiety, decreased functioning, racing thoughts, and perceptual changes. |
| Fava [8] | Relapse prodromes and residual symptoms | No | Medline and a manual search of the literature | 10^b^ | 20 subjects with BD (sample of unspecified age) | Manic prodromes were significantly longer than depressive prodromes | Semi-structured interview | Manic prodromes: increased activity, elevated mood, and decreased need for sleep.  Depressive prodromes: depressive mood, loss of energy, and difficult in concentration. |
| Howes et al [9] | Initial | No | PubMed, Medline, Embase, Embase Classic, PsycINFO | 14 | 3,733 subjects (children, adolescents, and adults with BD I, BD II, SAD, first episode of psychotic mania, people at high risk of psychosis, MD, psychotic NOS or brief psychotic episode) | 20.9 weeks–1.7 years in patients who developed psychotic mania  1.9 years in patients who developed non-psychotic mania | Ad hoc self-report questionnaire, ad hoc semi-structured rating scale, ad hoc symptom checklist, BPSS–R, IMPQ, BPRS, YMRS, SCL–90–R, HYP, K–SADS, CHT, CDI, SOPS, BDI, BAI | Initial prodromes: depressive mood, mood lability/swings, anxiety, racing thoughts, irritability/anger, and physical agitation. |
| Jackson et al [10] | Relapse | No | Medline, Best Evidence, PsycLIT, CINAHL, Embase, Cochrane Database of Systematic Reviews, PreMEDLINE | 17^c^ | 1,191 subjects (samples of different ages with bipolar and unipolar disorders) | Manic prodromes: 1–120 days  Depressive prodromes: 2–365 days | n/a | Early symptoms of mania: sleep disturbance, psychotic symptoms, mood change, psychomotor change, appetite change, increase anxiety, and other symptoms.  Early symptoms of bipolar depression: mood change, psychomotor symptoms, increased anxiety, appetite change, suicidal ideas, sleep disturbance, and other symptoms. |
| Lam et al [11] | Relapse | No | n/a | 6 | 223 subjects (samples of unspecified age with BD) | Mania prodromal periods: 1–84 days  Depression prodromal periods: 2–365 days | Spontaneous recall of prodromes, 40-item checklist, semi-structured interview, BPRS | Prodromal symptoms in mania: decreased sleep, more goal directed behavior/more energy, irritability, increased sociability, racing thoughts /ideas going too fast, increased optimism/increased confidence, feeling important, and decreased concentration.  Prodromal symptoms in depression: loss of interest in people or activity, feeling sad or want to cry, interrupted sleep, low motivation/loss of energy, low self esteem/self-confident, negative thinking, loss of concentration, and not able to put worries or anxiety aside. |
| Leopold et al [12] | Initial | No | ISI-WoS | 20 | 6,065 subjects (children, youths, and adults with some of the following disorders: BD, SAD or MD; children and siblings of parents with BD) | n/a | Survey, medical chart review, parental report, retrospective interview, retrospective semi-structured parental interview, retrospective semi-structured interview, BPSS–P, DES | Early symptomatology including changes in sleep and circadian rhythm, changes in mood, mood swings/affective lability, fearfulness/anxiety, and dissociative symptoms. |
| Malhi et al [13] | Initial | No | Medline, WoS, and a hand search of relevant literature | 28 | An unspecified number of offspring of parents with BD I and BD II, and community samples in childhood that were prospectively followed into adulthood. | n/a | Retrospective questioning of patients, prospective questioning of patients at risk, structured clinical interviews | Mood features: depressive symptoms/episodes, manic symptoms/subthreshold mania, mood lability, and cyclothymic temperament/cyclothymia.  Non mood features: irritability, anxiety/worry, energy changes (increased/decreased), anger/aggressiveness, and sleep disturbances. |
| Sierra et al [14] | Relapse | No | Medline,  Embase, and a hand search of the literature | 10^d^ | 548 subjects (samples of unspecified age) | Manic prodromes: 1–120 days  Depressive prodromes: 2–365 days | Semi-structured interview, 40 item-questionnaire, open self-administered questionnaires, BPRS, ChronoRecord | Prodromes of mania: increased activity, elevated mood, decreased need for sleep, more talkative, racing thoughts, increased self-steem, distractibility, increased sex drive, increased expending, feeling very religious, irritability, alcohol abuse, and unusual thought content.  Prodromes of depression: depressed mood, loss of energy, concentration difficulties, negative thinking, decreased sleep, increased sleep, loss of interest in activities or people, weight loss, loss of appetite, alcohol abuse, feeling very religious, suicidal ideas, irritability, cognitive symptoms, low self-esteem, and conceptual disorganization. |
| Skjelstad et al [15] | Initial | No | PsycINFO, PubMed, Embase, British Nursing Index | 8 | 1,625 subjects (children, adolescents, and adults with BD I, BD II, BD–NOS, psychotic mania, and ADHD) | 1.8–7.3 years | Postal survey, symptoms checklist, medical records, clinical records, parental notes, school reports, 84-ítem symptom checklist, BPSS-R | Distal prodromes: irritability and aggressiveness, sleep disturbances, mania-type symptoms and signs, hyperactivity, anxiety-related symptoms and signs, mood swings, and depression-type symptoms and signs.  Proximal prodromes: crying, bold/demanding, shy/timid, quick temper, overly sensitive, stubborn, conduct problems, depressed mood, decreased energy/tired, labile/mood changes, worried/anxious, elevated/irritable mood, irritability/anger, guilt/self reproach, decreased sleep, more talkative, anger dyscontrol, tantrums, bedwetting, loses temper, trouble concentrating, decreased concentration/attention/memory, interrupts, difficulty getting to sleep/insomnia, extremely clingy, touchy, often awakens in the night, night terrors, periods of extreme sadness, does not listen, very rapid speech, overly talkative, racing thoughts, craving for carbohydrates, suicidal ideation, increased energy/goal-directed activities, reckless/dangerous behavior, suspiciousness, hallucinatory experiences, strange or unusual ideas, grandiose ideas, drop in school/work function, social isolation, oppositionality, anxiety/nervousness, and anhedonia. |
| Van Meter et al [16] | Initial and recurrent prodromes | No | PsycINFO, PubMed | 11^e^  10^f^ | 1,084 subjects (range of averages ages = 10.6–43.85 years)^e^  1,000 subjects (range of averages ages = 12.0–47.2 years)^f^. The subjects had BD I and BD II | Manic episodes: 3.1–18.8 months^e^ Depressive episodes: 10.3 +/- 17.8 months^e,g^  Manic episodes: 0.5–1.3 months^f^ | IMPQ, BPSS-R, retrospective review or records, Children and Adolescent Research Evaluation, Clinical Interview, National DMDA questionnaire, semi-structured interview for mood swings, The Symptom Checklist, assessment and documentation of psychopathology^e^.  Two specific questions, ad hoc symptom checklist, Coping With Prodromes Interview, clinical interview, 40-item checklist for prodrome symptoms, open-ended survey question^f^ | Prodromal symptoms before an initial mood episode: too much energy, diminished ability to think, indecisiveness, pressured speech, talkative, elated mood, academic or work difficulties, insomnia, depressed mood, over-productive/goal directed, agitation, rage attacks, grandiosity, racing thoughts, anxiety, decreased need for sleep, irritable mood, fatigue, distractibility, sleep disturbance, disinhibited, weight loss/loss of appetite, hyperactive, suicidal thoughts, feeling of worthlessness, mood lability, delusions, appearance (unkempt, bizarre), guilt, auditory hallucinations, loss of interest, somatic complaints, sensitive, hypersexuality, flight of ideas, hypersomnia, weight gain, self-harm, suicide attempts, and visual hallucinations.  Prodromal symptoms before an initial manic episode: too much energy, talkative, racing thoughts, elated mood, decreased need for sleep, irritable mood, hyperactive, and overproductive/goal-directed^h^.  Recurrent prodromes: too much energy (occurred in more than half of the participants before a recurrent mood episode). |
| *Note.* PRISMA = Preferred Reporting Items for Systematic Reviews and Meta–Analyses; *k* = number of studies selected; ^a^ = only 5 studies attempt to describe the symptoms and behaviors characterizing the initial prodromal period; BD I= Bipolar Disorder type 1; BD II= Bipolar Disorder type 2; MD = Major Depression; n/a = not available; ^b^ = only one study reported depressive and manic prodromes in BD; SAD = Schizoaffective Disorder; NOS = Not Otherwise Specified; BPSS–R = Bipolar Prodrome Symptom Scale–Retrospective; IMPQ = Initial Mania Prodrome Questionnaire; BPRS = Brief Psychiatric Rating Scale; YMRS = Young Mania Rating Scale; SCL–90–R = Symptom Checklist–90–Revised; HYP = Hypomanic Personality Scale; K–SADS= Schedule for Affective Disorders and Schizophrenia for School Aged Children; CHT = Cyclothymic-Hypersensitive Temperament Questionnaire; CDI = Children’s Depression Inventory; SOPS = Scale of Prodromal Symptoms; BDI = Beck Depression Inventory; BAI = Beck Anxiety Inventory; ^c^ = 12 studies reported depressive and manic prodromes in BD; BPSS–P = Bipolar Prodrome Symptom Scale – Prospective; DES = The Dissociative Experience Scale; ^d^ = the ten studies offer information about prodromes or their duration; ADHD = Attention Deficit Hyperactivity Disorder; ^e^ = initial prodromes; ^f^ = recurrent prodromes; ^g^ = only one study reported on the prodrome duration before an initial depressive episode; DMDA = Depressive and Manic-Depressive Association; ^h^ = only five studies reported prodromes preceding an initial manic episode. | | | | | | | | |
