# Supplementary material for: Initial and relapse prodromes in adult patients with episodes of bipolar disorder: A systematic review
Source: Eur Psychiatry. 2020 Feb 12;63(1):e12. doi: 10.1192/j.eurpsy.2019.18 (PMC7315869; doi:10.1192/j.eurpsy.2019.18)
Supplement: Supplementary file 1 [file S092493381900018Xsup.zip › S092493381900018Xsup003.docx]

**Supplementary Table S3.** Modified Newcastle-Ottawa risk of bias scoring guide (based in Rotenstein et al. 2016)

| 1. Sample representativeness:   1 point: population recruited from multiple centers.  0 points: population recruited from a single center. |
| --- |
| 1. Sample size:   1 point: sample size was greater than or equal to 30 participants.  0 points: sample size was less than 30 participants. |
| 1. Non-participants:   1 point: the patient sample was consecutive. The characteristics of the participants and non-participants were compared.  0 points: the sample was of convenience. The characteristics of the participants and non-participants were not compared, or the comparison was insufficient. |
| 1. Assessment of prodromal symptoms in patients with bipolar disorder:   1 point: the study employed a commonly used measurement tool with appropriate psychometric properties (e.g., Bipolar Prodrome Symptom Scale-Retrospective, Early Warning Signs checklists).  0 points: the study only employed an ad hoc or infrequently used measurement tool without information about its appropriate psychometric properties (e.g., ad hoc interview, open-ended questions). |
| 1. Quality of descriptive statistics reporting:   1 point: the study reported descriptive statistics to describe the sociodemographic and clinical features (e.g., age, sex, education level, comorbidity, bipolar disorder subtype) with proper measures of dispersion (e.g., mean, standard deviation).  0 points: the study did not report descriptive statistics, incompletely reported descriptive statistics, or did not report measures of dispersion. |
| \| *Note.* The individual components listed above are summed to generate a total modified Newcastle-Ottawa risk of bias score for each study. Total scores range from 0 to 5. The quantitative selected studies were judged to be at low risk of bias (≥3 points) or high risk of bias (<3 points). \| \| --- \| |
