# Supplementary material for: Initial and relapse prodromes in adult patients with episodes of bipolar disorder: A systematic review
Source: Eur Psychiatry. 2020 Feb 12;63(1):e12. doi: 10.1192/j.eurpsy.2019.18 (PMC7315869; doi:10.1192/j.eurpsy.2019.18)
Supplement: Supplementary file 1 [file S092493381900018Xsup.zip › S092493381900018Xsup004.docx]

| **Supplementary Table S4.** Assessment of the risk bias of the studies selected in this systematic review | | | | | | |
| --- | --- | --- | --- | --- | --- | --- |
| Newcastle-Ottawa risk of bias scores (based in Rotenstein et al., 2016) for twenty quantitative studies | | | | | | |
| First author | Sample representativeness | Sample size | Non-participants | Assessmennt of prodromal symptoms | Quality of descriptive statistics reporting | Total |
| Estey et al. [4] | 1 | 1 | 0 | 1 | 1 | 4 |
| Hirschfeld et al. [33] | 1 | 1 | 0 | 0 | 1 | 3 |
| Noto et al. [36] | 0 | 1 | 0 | 1 | 1 | 3 |
| Özgürdal et al. [37] | 1 | 0 | 0 | 1 | 1 | 3 |
| Zeschel et al. [38] | 1 | 1 | 0 | 1 | 1 | 4 |
| Altman et al. [39] | 0 | 0 | 0 | 1 | 1 | 2 |
| Bauer et al. [40] | 1 | 1 | 0 | 1 | 1 | 4 |
| Goossens et al. [42] | 1 | 1 | 0 | 0 | 1 | 4 |
| Houston et al. [23] | 1 | 1 | 0 | 1 | 1 | 4 |
| Keitner et al. [24] | 1 | 1 | 0 | 0 | 1 | 3 |
| Lam et al. [25] | 1 | 1 | 0 | 1 | 1 | 4 |
| Lobban et al. [26] | 1 | 1 | 0 | 1 | 1 | 4 |
| Mander [27] | 0 | 0 | 0 | 0 | 1 | 1 |
| Mantere et al. [28] | 1 | 1 | 1 | 0 | 1 | 4 |
| Molnar et al. [29] | 0 | 0 | 0 | 0 | 1 | 1 |
| Perlman et al. [30] | 1 | 1 | 1 | 1 | 1 | 5 |
| Ryu et al. [31] | 0 | 1 | 0 | 0 | 1 | 2 |
| Sahoo et al. [32] | 0 | 1 | 1 | 0 | 1 | 3 |
| Smith et al. [34] | 1 | 0 | 0 | 0 | 1 | 2 |
| Wong et al. [35] | 1 | 1 | 0 | 0 | 1 | 3 |
| Responses in the Critical Appraisals Skills Program (CASP) checklist for two qualitative studies | | | | | | |
| First author | | | | | | |
| Benti et al. [22] | Yes = 6; Can't tell = 1; No = 2. Valuable research | | | | | |
| Fletcher et al. [41] | Yes = 7; Can't tell = 1; No = 1. Valuable research | | | | | |
